# MicroRNA-567 inhibits cell proliferation, migration and invasion by targeting FGF5 in osteosarcoma

**DOI:** 10.17179/excli2017-932

**Published:** 2018-01-15

**Authors:** Daodong Liu, Chaoju Zhang, Xiaolin Li, Hongmei Zhang, Qixiong Pang, An Wan

**Affiliations:** 1 Department of Orthopaedics, Jingzhou Hospital of Traditional Chinese Medicine, The Third Clinical College of Yangtze University, Hubei, China; 2 Department of Orthopaedics, Medical School of Yangtze University, Hubei, China; 3 Department of Orthopaedics, Wangjing Hospital of China Academy Chinese Medical Science, Beijing, China

**Keywords:** osteosarcoma, miR-567, FGF5, proliferation, migration and invasion

## Abstract

MicroRNAs (miRNAs) have been widely reported to have important regulatory roles in various human tumors, including osteosarcoma (OS). The aim of this study was to focus on the role of less well-known miRNA-567 (miR-567) in OS. We found the expression of miR-567 was significantly reduced in OS tissues and cell lines (MG-63, U2OS and Saos-2) compared with the adjacent normal tissues and normal osteoblastic cells (hFOB), respectively. Moreover, exogenous miR-567 overexpression inhibited OS cell proliferation, migration and invasion by CCK-8, Transwell assays, respectively. We further explored the mechanism underlying the suppressive effects of miR-567 on OS cells and identified a potential target of miR-567 binds to the 3'UTR of fibroblast growth factor 5 (FGF5) using TargetScan program. Furthermore, enforced expression of miR-567 decreased the expression of FGF5 in both MG-63 and U2OS cells using luciferase reporter assay and Western blotting. We also showed that overexpression of FGF5 could partially antagonize the suppressive effects of miR-567 on OS cell proliferation, migration and invasion. Taken together, our data indicated that miR-567 may function as a tumor suppressor by negatively regulating FGF5 and be potential therapeutic targets for the treatment of OS.

## Introduction

Osteosarcoma (OS) is the most common primary malignant skeleton tumor that derives from mesenchymal cells formed by primitive bone (Ottaviani and Jaffe, 2009[21]). The classic type of OS is rare, accounting for 0.2 % of all malignancies with an annual incidence of 3 per 1000,000 population (Picci, 2007[22]). OS predominantly occurs at the metaphyseal ends of the long bones of the extremities and is very infrequent in the soft tissues (Merimsky et al., 2004[19]). The prevalence of OS revealed a bimodal age distribution, with a larger peak around children and young adults, and a second peak in the elderly (Huh et al., 2012[13]; Sadoghi et al., 2010[24]). Few decades ago, patients usually undergo surgical amputation, however, once fewer than 20 % of them were completely cured (Ferguson and Goorin, 2001[9]). Nowadays, preoperative chemotherapy, wide local resection with careful pathologic, and adjuvant postoperative chemotherapy according to the rates of tumor necrosis have been considered the standard treatment for OS patients, but prognosis remains poor with less than 30 % survival in OS patients (Provisor et al., 1997[23]). Therefore, it is urgently to elucidate the pathology and molecular mechanism to better understand the progression of OS.

Recently, accumulating reports have focused on the impact of microRNAs (miRNAs) on the initiation, carcinogenesis and metastasis of cancer (Farazi et al., 2013[8]). MiRNAs are small non-coding RNAs with 20-23 nucleotides length that bind to the 3'-untranslated region (UTR) of their target mRNAs to repress translational activity and destabilize mRNA (Eichhorn et al., 2014[5]). Many preclinical studies revealed altered expression profiles of large quantity of miRNAs in a variety of cancers (Hayes et al., 2014[12]). Exploration of miRNAs function in OS pathogenesis and development is vital based on their possible role in the modulation of genes associated with proliferation, differentiation, apoptosis, and progression of cancer cells (Tufekci et al., 2014[27]). A previous study showed that miR-567 expression is aberrant in breast cancer, and ectopic expression of miR-567 impeded MDA-MB-231 cells proliferation and migration (Bertoli et al., 2017[2]). Shao et al. (2017[25]) pointed out that miR-567 can be used for a predictive marker in non-small-cell lung cancer diagnosis. In addition, a high frequency of miR-567 mutation was found in colorectal cancer cells and primary tumors (El-Murr et al., 2012[6]). However, the function of miR-567 in regulating OS development and progression are still unknown. 

Fibroblast growth factors (FGFs) are a family of polypeptides that play essential roles in metabolism and fundamental developmental processes of all multicellular organisms (Itoh and Ornitz, 2011[15]). Specially, abnormal FGFs expression and inopportune FGF receptors activation are implicated in uncontrolled cell proliferation, oncogenesis, skeletal development and diverse pathologic conditions (Teven et al., 2014[26]). Previous study has shown that murine FGF5 is typically expressed in the mesenchyme and skeletal muscle of the extremities, triggers mesenchymal fibroblasts proliferation and prevents skeletal muscle development (Clase et al., 2000[4]). Moreover, FGF5 is a direct target of miR-188-5p in hepatocellular carcinoma (HCC) and acts as an oncogene in HCC and glioblastoma multiforme (Allerstorfer et al., 2008[1]; Fang et al., 2015[7]). 

In the current study, we attempt to determine the expression and biological function of miR-567 in the OS. As a result, miR-567 was significantly downregulated in OS tissues and cell lines by using RT-PCR. Enhanced expression of miR-567 inhibited the proliferative, migratory and invasive potential of OS cells. In particular, FGF5 is a putative target of miR-567 as predicated by TargetScan and luciferase reporter assay. Our study provides the basis for using miR-567 as a novel therapeutic target in OS.

## Materials and Methods

### Human tissue samples

A total of 20 primary OS tissues and matched adjacent non-tumorous tissues were collected patients with OS from Jingzhou Hospital of Traditional Chinese Medicine (Jingzhou, China) and written informed consent was obtained from all patients. All the tissue samples were obtained after surgery resection and immediately snap-frozen in liquid nitrogen before use. None of the OS patients had received radiation therapy or chemotherapy prior to surgery. The present study was approved by the Ethics Committee of The Third Clinical College of Yangtze University (Hubei, China).

### Cell line culture

Human OS cell lines (U2OS, MG-63 and Saos-2) and the hFOB normal human osteoblast cell line were purchased from the American Type Culture Collection. All cell lines were cultured in the Dulbecco's Modified Eagle's Medium (DMEM, Thermo Fisher Scientifc, Inc.) supplemented with 10 % fetal bovine serum (FBS, Gibco, Grand Island, NY.) and maintained in a humidified atmosphere containing 5 % CO_2_ at 37 °C.

### Cell transfection

MiR-567 mimics and oligonucleotide scramble were obtained from the GenePharm (Shanghai, China). The pcDNA3.1-FGF5 plasmid and control vector were purchased from Amspring (Changsha, China). For miR-567 overexpression, miR-567 mimics were transfected into MG-63 and U2OS cells, while oligonucleotide scramble were used as a negative control. For restoration of FGF5 expression, pcDNA3.1-FGF5 plasmid was used to transfect the miR-567-overexpressing MG-63 and U2OS cells, while control vector was used as control. All cell transfection was performed using Lipofectamine 2000 (Invitrogen, CA, USA) according to the manufacturer's instruction. After 48 h of transfection, cells were used for the following *in vitro* experiments.

### Quantitative RT-PCR (qRT-PCR)

Total RNA was extracted with TRIzol (Invitrogen, USA) and then converted into cDNA using the M-MLV (Promega, USA) according to the manufacturer's instructions. Quantification for miR-567 was performed on ABI 7900 system (Applied Biosystems) using miRNA qRT-PCR kit and miR-567 primers (Exiqon, Euroclone, Italy). The relative expression of miR-567 was determined for tissue samples and cell lines using 2^-ΔΔCT^ method (Livak and Schmittgen, 2012[17]). The expression of U6 was used as the internal control. Experiments were performed three times in triplicate.

### Western blot analysis

Proteins were isolated from transfected cell lines using a RIPA lysis buffer (Sigma, USA). Then the protein concentration was determined by a bicinchoninic acid protein assay kit (Beyotime Institute of Biotechnology, Inc.) following the manufacturers' instruction. Equal protein was separated by 12 % SDS-PAGE and then transferred to polyvinylidene fluoride (PVDF) membranes (Millipore, USA). After blocked with 5 % skimmed milk in PBS for 1 h at room temperature, the membrane was incubated with antibodies against Ki-67, PCNA, E-cadherin, Vimentin, FGF5 and GAPDH at 4 °C overnight. Subsequently, the membranes were washed with PBS for three times, followed by incubation with horseradish peroxidase (HRP)-conjugated secondary antibodies for 2 h at room temperature. After washing with PBS three times, the membrane was visualized by ECL Plus reagents (Beyotime, China).

### Cell proliferation

Transfected MG-63 or U2OS cells were separately seeded in 96-well plates at a density of 4 × 10^3^ cell per well. Three duplicate wells were set for each group. At 24, 48, 72 and 96 h incubation, 10 μl of CCK-8 reagent was added into each well, followed by 4 h incubation. The optical density (OD) was measured by Microplate Reader (Bio-Rad, USA) at the wavelength of 450 nm.

### Cell migration and invasion

To analyze the migratory capacity of transfected cells, approximately 5×10^4^ cells were seeded into the upper chamber of 8 μm transwell inserts (BD Biosciences, US) in the serum-free media and incubated for 24 h at 37 °C. Then FBS-containing medium was added in the lower chamber as a chemoattractant. Non-migratory cells in the upper chamber were removed carefully and cells that migrated to the lower chamber were fixed in 20 % methanol and stained with 0.1 % crystal violet. Next, the stained cells were viewed under an inverted microscope (Nikon, Chiyoda-Ku, Japan). For the transwell invasion assay, all procedures were the same as the transwell migration assay, except that the upper chamber was coated with matrigel (BD Biosciences, US). For migration and invasion assays, five randomly selected fields were quantified and the average number of cells was calculated.

### Dual-luciferase reporter assay

The putative targeting gene (FGF5) of miR-567 was predicted using TargetScan software (http://www.targetscan.org) and verified by dual-luciferase reporter gene assay. Briefly, the site-directed mutagenesis kit (Stratagene, USA) was used to construct mutant FGF5 3' UTR. FGF5 3' UTR and mutant FGF5 3'UTR were inserted into pGL4.13 vectors (Promega, Madison, USA) and named as wild type (WT) or mutated (MUT) pGL-FGF5-3'UTR, respectively. All recombined plasmids were verified by DNA sequencing. For luciferase reporter assays, MG-63 and U2OS were cultured in 48-well plates and co-transfected with a mixture of WT or MUT plasmids and miR-567 mimics or scramble mimics using Lipofectamine 2000. After 48 h transfection, luciferase activity was measured using the dual-luciferase reporter assay system according to the manufacturers' instructions (Promega, USA). Reporter gene expression was expressed as the ratio of Firefly luciferase activity to Renilla luciferase activity.

### Statistical analysis

All statistical analysis was performed using GraphPad Prism software version 5.0 (GraphPad, Software, Inc., La Jolla, CA, USA). Quantitative data are expressed as mean ± SD of at least three experiments. For comparisons, ANOVA or Student's t-test was used to evaluate difference between more than two groups or two groups, respectively. The *p*-value of less than 0.05 was considered statistically significant.

## Results

### The expression of miR-567 was downregulated in OS tissues and cell lines 

**Figure 1:**
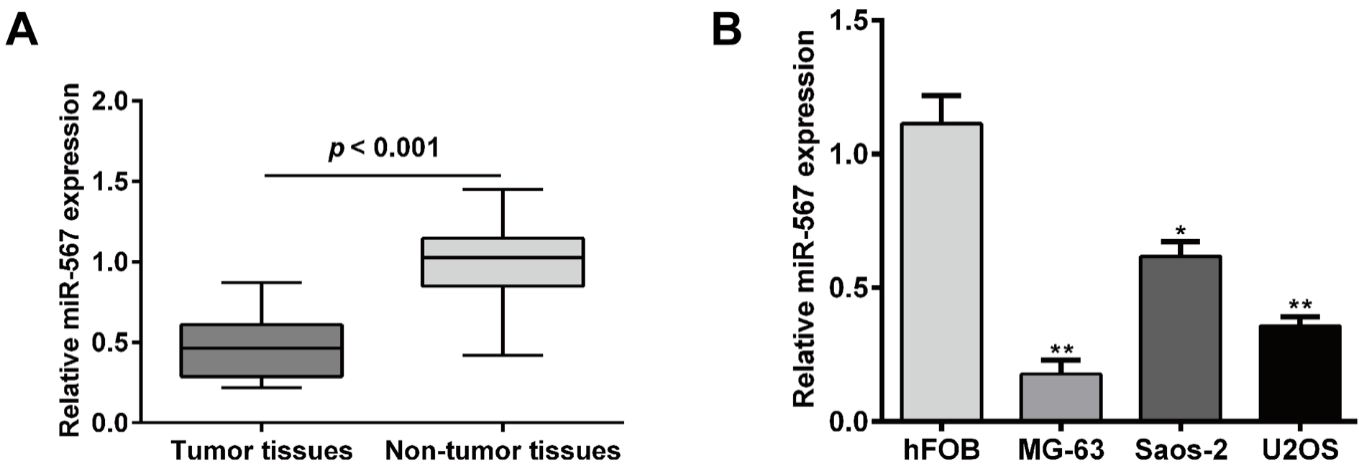
miR-567 expression levels were downregulated in osteosarcoma tissues and cell lines. (A) Relative expression levels of miR-567 were determined in 20 osteosarcoma patient tissues samples and adjacent non-tumor tissues using quantitative real-time PCR analysis. (B) Relative expression of levels of miR-567 were measured in MG-63, U2OS and Saos-2 cell lines and normal hFOB cell line using quantitative real-time PCR analysis. Each assay was conducted in triplicate and repeated three times. The data show the mean of triplicate repeats ± SD. *p < 0.05, **p < 0.01 *vs.* normal hFOB cell line

We first performed qRT-PCR to determine the expression of miR-567 in a total of 20 OS tissues and matched adjacent non-tumor tissues. As shown in Figure 1A(Fig. 1), miR-567 expression levels in OS tissues were significantly downregulated compared to those in the adjacent non-tumor tissues (*p* < 0.001). In addition, the expression of miR-567 further confirmed in several common OS cell lines, including MG-63, U2OS and Saos-2. Consistently, miR-567 expression levels were significantly downregulated 0.84 ± 0.14 in MG-63 (*p* < 0.01), 0.68 ± 0.21 in U2OS (*p* < 0.01) and 0.45 ± 0.28 -fold in Saos-2 (*p* < 0.05) cell lines compared with normal human osteoblast cell line hFOB, respectively (Figure 1B(Fig. 1)). These data suggest miR-567 might play the key roles in the tumor progression of OS.

### Elevated expression of miR-567 suppressed the OS cell proliferation

**Figure 2:**
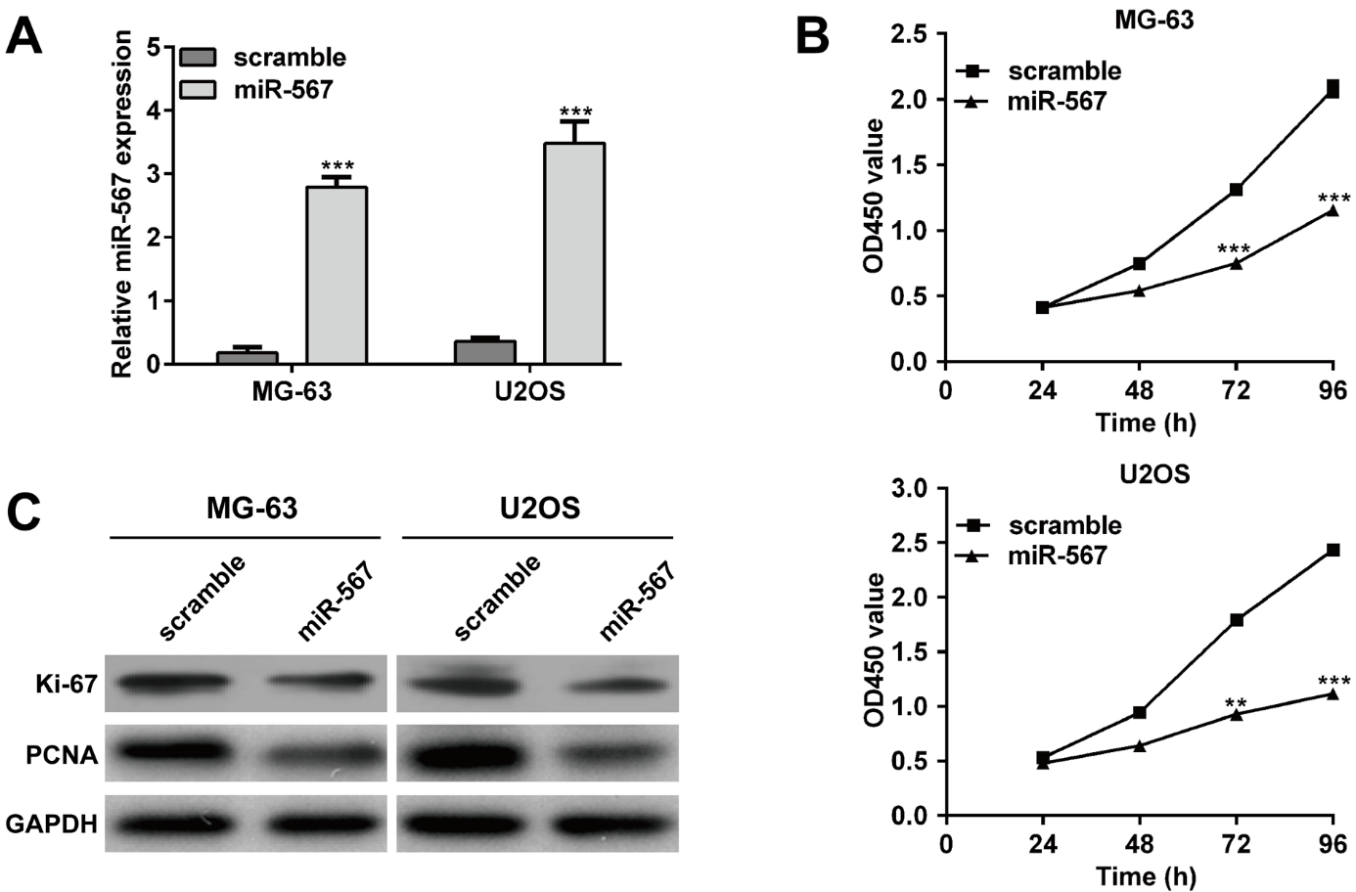
Elevated expression of miR-567 suppressed the osteosarcoma cell proliferation. (A) Quantitative real-time PCR analysis was performed to determine the relative expression of miR-567 in MG-63 and U2OS cells transfected with miR-567 mimics or scrambled plasmid. (B) CCK-8 assay was used to evaluate cell proliferation ability in MG-63 and U2OS cells transfected with miR-567 mimics or scrambled plasmid. (C) The protein expression levels of Ki-67 and PCNA were detected in MG-63 and U2OS cells transfected with miR-567 mimics or scrambled plasmid using Western blot. Each assay was conducted in triplicate and repeated three times. The data show the mean of triplicate repeats ± SD. GAPDH was used as the internal control. **p < 0.01, ***p < 0.001 *vs.* scrambled group

To investigate the function of miR-567 on OS development, MG-63 and U2OS with lower miR-567 expression were selected for transfection with miR-567 mimics or scramble plasmid. As shown in Figure 2A(Fig. 2), transfection with miR-567 mimics led to a significant increase in miR-567 expression levels compared with those in scramble control group, confirmed by qRT-PCR in both MG-63 and U2OS cells (*p* < 0.001). CCK-8 assay indicated that ectopic expression of miR-567 suppressed the MG-63 (*p* < 0.001) and U2OS (*p* < 0.01, *p* < 0.001) cell proliferation (Figure 2B(Fig. 2)). Moreover, overexpression of miR-567 decreased the Ki-67 and PCNA protein expression both in the MG-63 and U2OS cells (Figure 2C(Fig. 2)). 

### Elevated expression of miR-567 inhibited OS cell migration and invasion

**Figure 3:**
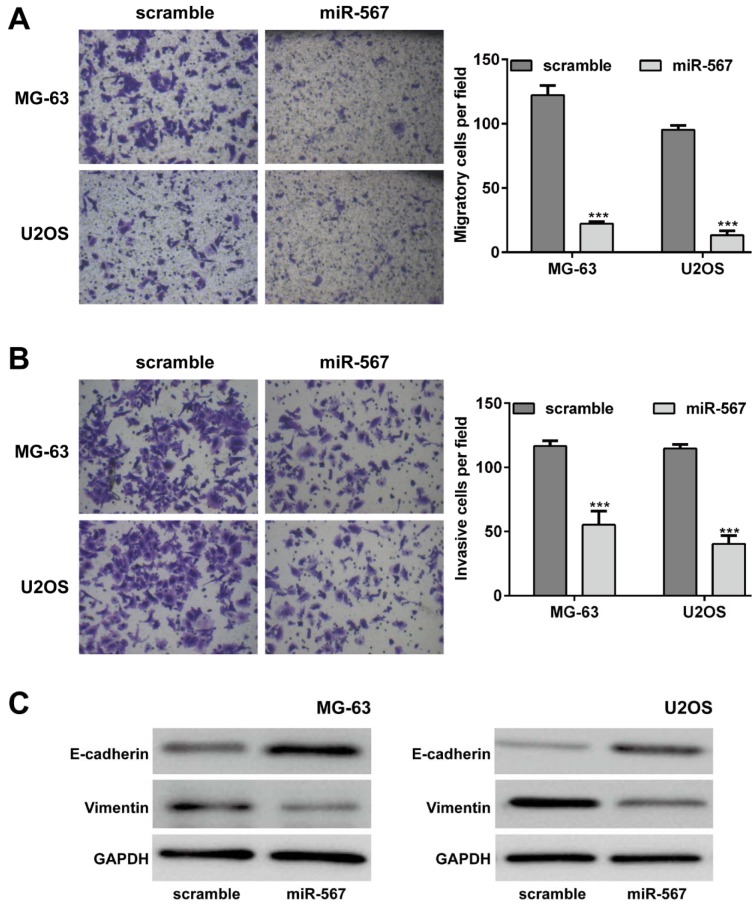
Elevated expression of miR-567 suppressed the osteosarcoma cell migration and invasion ability. Transwell assay were performed to examine the cell (A) migration and (B) invasion in MG-63 and U2OS cells transfected with miR-567 mimics or scrambled plasmid. (C) The protein expression of E-cadherin and Vimentin was determined by Western blot in MG-63 and U2OS cells transfected with miR-567 mimics or scrambled plasmid. Each assay was conducted in triplicate and repeated three times. The data show the mean of triplicate repeats ± SD. GAPDH was used as the internal control. ***p < 0.001 *vs.* scrambled group

To further evaluate the function of miR-567 on the migration and invasion capability, Transwell chamber assay was performed in MG-63 and U2OS cells. The results of Transwell experiments indicated that upregulation of miR-567 could restrict the migratory and invasive ability of MG-63 and U2OS cells (Figure 3A and B(Fig. 3),* p* < 0.001). The average number of migratory cells in the miR-567 mimics group was significantly less than that in the scramble group (MG-63: 22.3 ± 1.5 *vs.* 122.3 ± 7.4;U2OS: 13.3 ± 3.5 *vs.* 95.3 ± 3.2). As for cell invasion, the number of invasive cells was reduced from 116.7 ± 4.2 in miR-567 mimics group to 55.3 ± 10.8 in scramble group in MG-63. Similarly, upregulation of miR-567 remarkably decreased the number of invasive cells in U2Os cells.

Moreover, we found ectopic expression of miR-567 increased the epithelial marker E-cadherin protein expression and decreased the mesenchymal marker Vimentin protein expression in both MG-63 and U2OS cells (Figure 3C(Fig. 3)).

### FGF5 was a direct target gene of miR-567

**Figure 4:**
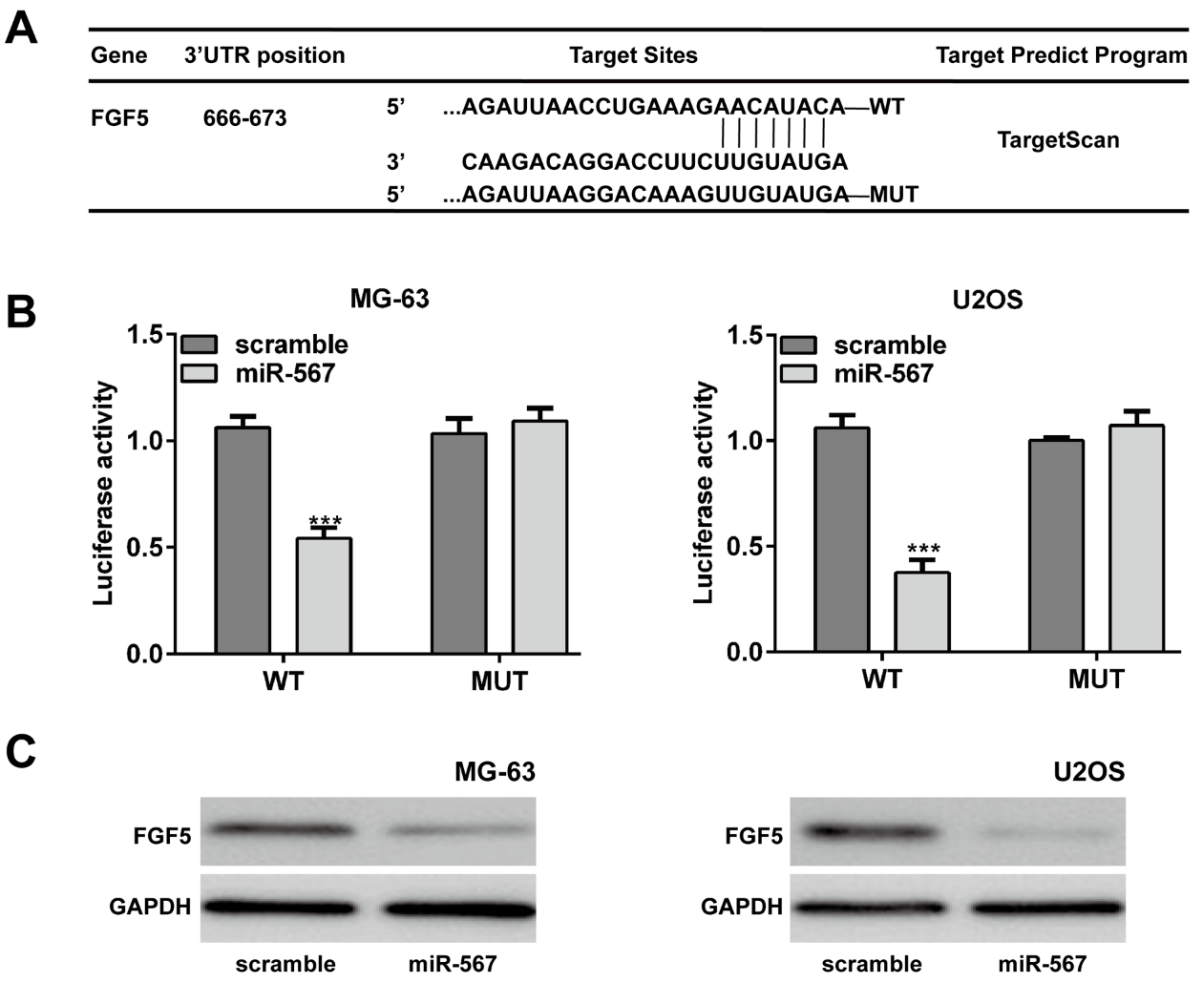
FGF5 was a direct target gene of miR-567 in osteosarcoma cells. (A) The putative binding site of miR-567 and FGF5 is shown. (B) Luciferase report assay demonstrated that miR-567 overexpression suppressed the luciferase activity of wild-type (WT) 3ʹUTR of the FGF5 vector in the MG-63 and U2OS cells. (C) Ectopic expression of miR-567 decreased the FGF5 protein expression in the MG-63 and U2OS cells by Western blot. Each assay was conducted in triplicate and repeated three times. The data show the mean of triplicate repeats ± SD. GAPDH was used as the internal control. ***p < 0.001 *vs.* scrambled group

According to the prediction by TargetScan database (http://www.targetscan.org), FGF5 could be one of the target genes of miR-567 (Figure 4A(Fig. 4)). To verify this predication, luciferase reporter vectors containing WT and MUT fragments of the FGF5 3'-UTR were constructed. As shown in Figure 4B(Fig. 4), the luciferase reporter assay showed that only MG-63 and U2OS cells in the WT 3'UTR + miR-567 mimics group had a remarkably lower luciferase activity than other groups (*p* < 0.001), suggesting that miR-567 could restrain FGF5 translation by binding to its 3'UTR. Furthermore, overexpression of miR-567 suppressed the FGF5 protein expression both in the MG-63 and U2OS cells (Figure 4C(Fig. 4)).

### Enhanced expression of FGF5 could partially antagonize the suppressive effects of miR-567-overexpressing in OS cells

**Figure 5:**
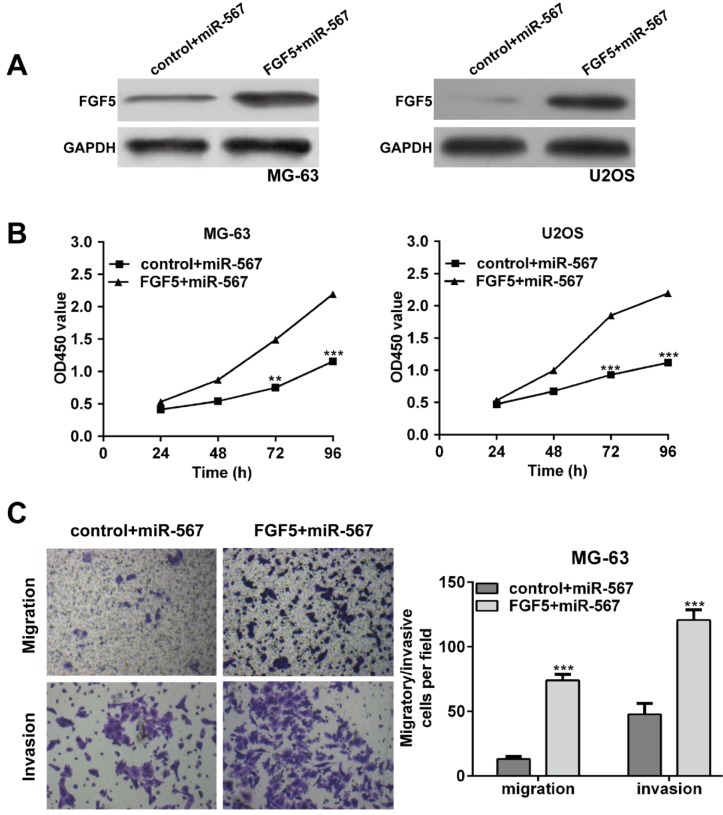
Overexpression of FGF5 partially rescues the inhibitory effects of miR-567 in osteosarcoma cells. MG-63 and U2OS cells were transfected with miR-567 mimics or co-transfected with miR-567 mimics and FGF5 plasmid. (A) Western blot analysis was performed to examine the protein levels of FGF5 in transfected MG-63 and U2OS cells. (B) CCK-8 assays were conducted to determine the proliferation rate of MG-63 and U2OS cells after 48 h transfection. (C) Transwell assay was used to determine cell migration and invasion in MG-63 cells after 48 h transfection. Each assay was conducted in triplicate and repeated three times. The data show the mean of triplicate repeats ± SD. GAPDH was used as the internal control. **p < 0.01, ***p < 0.001 *vs.* scrambled group

As FGF5 has been reported to be involved in multiple tumor cell proliferation and metastasis (Fang et al., 2015[7]; Hanada et al., 2001[10]), it was thus speculated that FGF5 might mediate the inhibitory effect of miR-567 on cell proliferation, migration and invasion in OS. To verify this hypothesis, we performed rescue experiments by transfecting pcDNA3.1-FGF5 plasmid into miR-567-overexpressing OS cells and examined cell function. After transfection, the protein level of FGF5 was significantly higher in the FGF5 + miR-567 group than that in the control + miR-567 group (Figure 5A(Fig. 5)).

CCK-8 assay indicated that overexpression of FGF5 partially rescued the impaired cell proliferation induced by miR-567 mimics in MG-63 and U2OS cells (Figure 5B(Fig. 5), *p* < 0.01, *p* < 0.001). In addition, we also found the migration and invasion ability of MG-63 cells were significantly increased in the FGF5 + miR-567 group when compared with those in the control + miR-567 (Figure 5C(Fig. 5), *p* < 0.001). These findings further demonstrated that FGF5, as a target gene of miR-567, could antagonize the suppressive effects of miR-567 on OS cell proliferation and invasion.

## Discussion

The key problem in the identification and characterization of the miRNA function is the comprehensively uncover its impact on the translational and transcriptional levels of target mRNA as well as the outcome in the cell biological behaviors (le Sage et al., 2007[16]). In the present study, we first show that miR-567 is poorly expressed in OS tissues and OS cell lines. Notably, our results revealed miR-567 as endogenous modulator of FGF5 and confirmed the importance of miR-567-FGF5 interaction for the proliferation, migration and invasion in OS cell lines.

Cell proliferation is a critical biological process frequently uncontrolled in tumors (Hanahan and Weinberg, 2000[11]). Here, enhanced expression of miR-567 exhibited a significant anti-proliferative effect in OS cells, MG-63 and U2OS. Additionally, Western blot analysis showed that miR-567 negatively regulated two cell proliferative markers Ki-67 and PCNA. The nuclear antigen Ki-67 is preferentially occuring during certain cell cycle phases G1, S, and G/M, but deficient in G0 (Inwald et al., 2013[14]). PCNA is a nuclear nonhistone protein that is produced in late G1 and S phases, while lack in quiescent cells, and required for DNA synthesis (Bologna-Molina et al., 2013[3]). Because miR-567 responsive genes Ki-67 and PCNA are known cell-cycle genes, one predication is that OS cells with miR-567 deficiency will promote cell proliferation by activating the cell cycle progression and DNA replication.

During cancer development, epithelial mesenchymal transition (EMT) is a crucial cellular process in the conversion of immotile epithelial cells into motile mesenchymal cells (Yang and Weinberg, 2008[29]). OS cells are known to undergo EMT-related pathological processes to possess an increased migration and invasive abilities (Yang et al., 2013[28]). EMT has a particular close relationship with invasive malignant neoplasms, which may supply insights into the pathological mechanism of OS. In a previous study, overexpression of miR-567 has been implicated in the suppression of breast cancer cell migration. Here, heightened expression of miR-567 also displayed an inhibitory effect on the migration as well as invasion in OS cells. Moreover, overexpression of miR-567 was shown to increase E-cadherin expression and reduce Vimentin expression. E-cadherin acts as a transmembrane glycoprotein that involved in epithelial cell-cell adhesion and EMT, while high level of Vimentin is positively associated with tumorigenesis (Nijkamp et al., 2011[20]). Our studies indicate that miR-567 functions as an inhibitor of EMT, which may at least partially though alteration of EMT biomarkers E-cadherin and Vimentin expression. Disturbance of EMT played a major role in the attenuation of miR-567-induced OS cell migration and invasion. 

Furthermore, FGF5 was predicted and demonstrated as the potential target gene of miR-567. Expression of FGF5 has been found to control osteoblast function and differentiation, and impair skeleton muscle development (Clase et al., 2000[4]; Marie, 2003[18]). In addition, FGF signaling exerts a biological effect on cellular behaviors by regulating the expression of various genes involved in RAS/MAP kinase, PI3/AKT, and/or PLCγ pathways. In this study, overexpression of FGF5 partially restores the impaired cell proliferation, migration and invasion induced by miR-567, supporting a key role of FGF5 signaling in these pathological processes. Simultaneous overexpression of miR-567 appears to have a negative effect in regulating FGF5, then resulted in a significant change in proliferation and EMT biomarkers.

In summary, our study indicated that miR-567 expression levels were reduced in OS tissues and cell lines. Enhanced expression of miR-567 have low proliferative, migratory and invasive capabilities though interfere translational levels of FGF5. Therefore, identification and characterization of the function of miR-567 could deepen our understanding of the pathological development of OS.

## Declaration of interest

The authors report no conflicts of interest. No external funding was sought or obtained to support the work described in the manuscript.
